# Arthroscopic Repair in Tibial Spine Avulsion Fractures Using Polyethylene Terephthalate Suture: Good to Excellent Results in Pediatric Patients

**DOI:** 10.3390/jpm11050434

**Published:** 2021-05-19

**Authors:** Octav Marius Russu, Tudor Sorin Pop, Emilian Ciorcila, István Gergely, Sándor-György Zuh, Cristian Trâmbițaș, Paul Gabriel Borodi, Zsuzsanna Incze-Bartha, Andrei Marian Feier, Vlad Alexandru Georgeanu

**Affiliations:** 1 Department of Orthopaedics and Traumatology, Clinical County Hospital, 540139 Tîrgu Mureș, Romania; octav@genunchi.ro (O.M.R.); sorintpop@yahoo.com (T.S.P.); gergelyistvan@studium.ro (I.G.); zuh.sandor@gmail.com (S.-G.Z.); c_trambitas@yahoo.com (C.T.); zsuzsanna.incze-bartha@umfst.ro (Z.I.-B.); andreifeier@gmx.com (A.M.F.); 2 Faculty of General Medicine, University of Medicine, Pharmacy, Sciences and Technology, 540139 Tîrgu Mureș, Romania; borodi.paul@yahoo.com; 3 Department of Anatomy and Embryology, University of Medicine, Pharmacy, Sciences and Technology, 540139 Tîrgu Mureș, Romania; 4 Clinic of Orthopaedic and Trauma Surgery, “St. Pantelimon” Hospital, 021659 Bucharest, Romania; vgeorgeanu@hotmail.com

**Keywords:** tibial spine, avulsion, arthroscopy, suture

## Abstract

Background: The objective of the arthroscopic treatment in tibial spine avulsion fractures (TSAF) is to achieve firm reduction and strong internal fixation while still having the patient undergo a minimally invasive procedure. Material and methods: The study was performed on 12 young patients with avulsion fracture of the anterior tibial spine. All 12 patients had type 3 Modified Meyers and McKeever fractures. The injury mechanism was direct anterior to posterior trauma in full leg length hyperextension with sport trauma reported in all cases. The physical examination revealed decreased range of motion, extension deficit, and pain during walking. Radiology, MRI, and CT pathologic findings described complete fracture of the anterior tibial spine with no clear signs of callus formation at the time of examination. All patients underwent arthroscopic suture surgical treatment. The Tegner, the Lysholm, and the International Knee Documentation Committee (IKDC) scores were used to evaluate subjective outcomes at three and six months after the surgery. Radiographs were used to assess callus formation and healing status of the fracture. Results: The mean IKDC score was 33.4 ± 23.3 (*p* = 0.032) preoperatively and 84.2 ± 14.3 at final follow-up (*p* = 0.0032, CI = 95%). The mean Tegner score improved from 3.8 ± 1.1 pre-operatively to 6.7 ± 2.2 at six months follow-up (*p* = 0.0231, CI = 95%). The Lysholm score differed significantly at baseline compared to final follow-up (53.7 ± 17.3 vs. 87.7 ± 9.9; *p* = 0.0066, CI = 95%). In all cases (*n* = 12), the radiographs taken after six months revealed the healing of the fracture in the anatomic position without secondary displacement. No functional knee instability was detected at the end of the study. Conclusions: The study provides preliminary promising results regarding fracture healing, knee stability, and functional subjective scores. Patient selection was a major factor of success prediction for this technique.

## 1. Introduction

Every year, more people between 1- and 44-years-old die from high energy trauma than from cancer or cardiovascular diseases. Tibial spine avulsion fractures (TSAF) rank third in children. In two thirds of cases, the fracture exerts influence only on tibia, while in the rest of the cases, the fibula is also involved. In pediatric patients, avulsion fractures are considered to be the result of the incomplete union of ossified tibial spines happening prior to an anterior cruciate ligament (ACL) rupture [1,2,3].

Joints are complex structures that enable repetitive, pain free, frictionless movements due to their very specialized structure. The immaturity of the musculoskeletal system, consisting in both the developing osteogenic cells and extracellular matrix, makes TSAF fractures the most common in children [4,5].

The mechanism of the fracture has not been fully understood yet. In the majority of the cases, the injury happens due to altitude fallings, motor vehicle injuries with the knee in hyperextension, and sports injuries (skiing, basket, soccer, horse riding, etc.). The findings of various studies show different results, due to the high variability in patient sample size and the habits of the population at that time. Historically, bicycle accidents were considered to be the most frequent cause of TSAF. In spiral fractures, the force mechanism that produces the fracture is almost every time represented by a torsional force, with the feet blocked to the ground. Transverse or comminuted fractures are mainly due to direct trauma. Complications such as: vascular lesions, nerve injuries, compartment syndrome premature physeal closure, and malunion are possible, but not often seen [6,7].

Meyers and McKeever were the first to classify these fractures in 1959 into three types. Type I fractures have little or no displacement of the fragment from its tibial location, type II fractures have the anterior 1/3 to 1/2 of the tibial eminence avulsed and hinged, and type III fractures involve full isolation of the segment [8]. With the occurrence of arthroscopy and magnetic resonance imaging, it is now known that associated soft tissue injuries are common with TSAF [9,10].

Most TSAF are treated with reduction under anesthesia and cast immobilization. The surgical treatment is advised when proper reduction cannot be achieved. Other possible conditions that will indicate the surgery are: open fractures, compartment syndrome, floating knee, unstable fractures, and fractures in patients with spasticity. The number of surgical procedures is increasingly high, despite the fact that conservative treatment has been a satisfying practice for many years. The explanation resides in the changes of lifestyle, increased sports participation in children, the financial system, and the patient’s desires [2]. 

Proper treatment should offer anatomic reduction of the fragment and operative techniques range from arthrotomy with screws, wires, staples, pins, or suture fixation to arthroscopically assisted internal fixation with cannulated or cortical screws, percutaneous pins, and suture fixation with and without hardware [10,11,12]. Our objective is to evaluate functional subjective and objective outcomes in pediatric patients that underwent arthroscopic suture of tibial eminence avulsion fractures and to describe the surgical technique used. 

## 2. Materials and Methods

This is a prospective clinical study that was led in the Department of Orthopaedics and Traumatology in Tîrgu Mureș, Romania. The study was authorized by the local ethical committee and performed in compliance with the principles of Good Clinical Practice and the Declaration of Helsinki concerning medical research on humans and the country-specific regulations. The following were chosen as inclusion criteria: male and female pediatric patients aged between 6 to 16 years old that presented in our department with avulsion-fractures of the tibial eminence corresponding to type III Meyers and McKeever, practiced any sports for >6 months period, and had no previous surgery on the affected knee. Exclusion criteria were the following: trauma history for >4 weeks; patients diagnosed with osteogenesis imperfecta, any type of vitamin deficiencies, or any history of skeletal dysplasias; and inactive patients. From 18 subjects that were screened and diagnosed with avulsion of the tibial spine, 6 had one or more exclusion criteria. All the interventions were performed by the same senior orthopedic-trauma surgeon. The followed-up of the patients has been done at 3 and 6 months postoperatively. Digital antero-posterior and latero-lateral radiographs of the tibiofemoral joint were obtained before surgery and at each follow-up visit. After arthroscopic reduction and fixation, the patients used a similar knee brace and were mobilized with no weight bearing for 4 weeks. After that, they began partial weight bearing and full weight bearing by 10 weeks. Soft quadriceps and hamstring exercises were initiated at 6 weeks postoperative. Data collection and patient evaluation were conducted by a study nurse under observation of the senior author.

### 2.1. Outcomes Assessment 

The International Knee Documentation Committee (IKDC) Questionnaire, Lysholm score, and Tegner activity scale were utilized to evaluate the results. They were assessed before surgery and at each scheduled follow-up attendance (3 and 6 months, respectively). Anteroposterior and latero-lateral radiographs were performed at each follow-up visit. The IKDC score underwent several studies for validity and responsiveness and has proven to be the most reliable patient-reported outcome measure in knee-related sports injuries [13]. After undergoing numerous and detailed studies in the past decades, the Lysholm score has proven acceptable psychometric parameters as a patient-administered score [14]. Eight subscales are evaluated in the up-to-date version (limp, support, locking, instability, pain, swelling, stair climbing, and squatting). The values from each subscale are summed in order to give a total normalized score [14,15]. Knee anterior instability was assessed using the Rolimeter device.

### 2.2. Surgical Technique

After obtaining suitable spinal or epidural anesthesia, the patient is put in a supine position. When general anesthesia is performed, the femoral block is considered useful for the control of the postoperative pain [16]. The procedure can be effectuated either with a leg holder or with the leg free. Tourniquets are used and inflated.

Visualization is set through a standard anterolateral portal with the intra-articular hematoma firstly evacuated through the standard anteromedial portal. Once the hematoma and debris are cleared and visualization is established, diagnostic arthroscopy is carried out to take inventory of the associated injuries.

The tibial spine fragment is verified to determine the amount of displacement, comminution, and other soft structures involvement. The anterior cruciate ligament should be inspected for ecchymosis and attenuation [17]. With the arthroscope in the anterolateral portal, a motor-powered shaver is used to debride the small cartilage and bone fragments, and any interfered soft tissue, from the place of fracture. The fracture afterwards is reduced using the anterior cruciate ligament reconstruction tibial guide, with the knee at 90 degrees of flexion through the anteromedial portal and oriented just medial to the fracture site.

A small skin incision (1–2 cm) is prepared over the proximal anteromedial tibia, the extra-articular portion of the guide is secured over the tibial cortex, and then a 2.0 mm guide wire is inserted through the anterior cruciate ligament reconstruction tibial guide to exit just medial to the fracture line. Afterwards, the medial tunnel is drilled by a 4.5 mm cannulated drill over the anteriorly placed guide wire. The same procedure is conducted on the lateral side of the fracture crater, with the camera in the anteromedial portal, and the lateral tunnel is drilled [17,18,19].

The anterior cruciate ligament tibial guide is placed back in the knee using the anteromedial portal, and the lead is just lateral to the fracture line [20,21]. When the correct intra-articular locus of the guide is settled, it is then fortified using the anterior incision of the skin on the anteromedial tibia, about 1 to 1.5 cm from the first hole; with a peak guidewire of 2.0 mm, a drill is made just lateral to the fracture site. The lateral tunnel is drilled by a 4.5 mm cannulated drill over the previous wire, and then a second thin piece of 0.8 mm stainless steel wire is passed into the joint lateral to the fracture line [15]. A sterile braided polyethylene terephthalate suture coated with silicone is used for final reduction and fixation. This suture is chosen for maximum security in fixation of tissues under stress conditions, permanent support, and strength of the material being retained long after hard callus formation. 

Arthroscopic wire suture technique offers the advantage of using only two portals, allows the cure of concomitant injuries, and there is no need for costly implants. The disadvantages of this technique consist of the difficulty in inserting the suture wire, the low tension, and not enough reduction. Furthermore, the full reduction of the fragment can be affected by the tunnels that can pass through the place of fracture (Table 1).

## 3. Results

There was no statistically significant difference between patients in regards to demographic data and patient characteristics (Table 2).

The preoperative IKDC score improved from 33.4 (range 27–61) preoperatively to 84.2 (range 70–97) at the final follow-up (*p* = 0.0032). The mean Tegner score improved from 3.8 (range 3–9) pre-operatively to 6.7 (range 4–9) at 6 months follow-up (*p* = 0.0231). At the three-month follow-up, there was no significant difference in the mean Tegner score compared to preoperative scores. The Lysholm score improved from the baseline, with 53.7 (range 33–64) before the surgery and 87.7 (range 72–97) at the final follow-up (*p* = 0.0066). Outcomes recorded before and at each follow-up are represented in Table 3. In all patients (*n =* 12), the radiographs taken after 6 months showed healing of the fracture in the anatomic position with no secondary displacement, and none of the knees displayed clinical or radiological proof of degenerative disease. None of the patients had functional knee anterior instability when tested with the Rolimeter. 

## 4. Discussion

The findings of this study prove that arthroscopic repair in TSAF using polyethylene terephthalate suture has good to excellent results in pediatric patients, with significant improvements in the IKDC, Tegner, and Lyshold scores as well as in the radiographs taken after 6 months. 

This technique has been reported to provide good to excellent outcomes [15]. Having only two portals and being able to arthroscopically assess and handle the associated lesions transformed this technique into a gold standard for avulsion fractures of the tibial eminence [17,18]. The economic burden is avoided due to no extra-instruments being required [15,19]. However, the drill of the tunnels should be carefully assessed as they may pass through the fracture line and influence the final outcome [15]. Furthermore, unexperienced surgeons may also encounter difficulties inserting the loops and cause low to no tension or a fragile fixation [15,22]. The multi-strand braided suture composed of polyethylene terephthalate, which gives it superior strength and abrasion resistance, makes it a good choice in orthopedic surgery. Suture breakage during knot tying is low, which is important in arthroscopic procedures. 

TSFA implies a tear of the ACL complex. Any remanent displacement can lead to knee laxity and functional impairment. It has been demonstrated that anatomically reduced fractures have a disposition to displace with time. Taking this into consideration, the reduction and fixation of all type II, III, and IV fractures is advised [23,24,25]. There is no agreement regarding the type of fixation that is most beneficial for these fractures; the existing techniques at hand range from excision to K-wire to screw to absorbable sutures, or, more recently, suture anchors or meniscal arrows. Both sutures and screw fixation methods have been worked in cadavers. Bong et al. described that the initial ultimate strength was higher with three no.2 fiber wire sutures than with a 4 mm × 40 mm partially threaded cannulated screw with a washer, whereas Eggers et al., in a porcine model, discovered that suture fixation offers better resistance than screw fixation under cyclic loading [12,26]. Hunter and Willis [24] showed no significant difference in outcomes regarding the type of fixation, whereas Seon et al. reported that both the screws and suture fixation ways produced a relatively good result considering the functional outcome and stability [27,28]. 

The suture features that are significant for a solid construct include strength and knot security. Gerber et al. observed that a nonabsorbable suture is firmer and stiffer than an absorbable suture. Lo et al. then found that FiberWire is stronger than Ethibond. Strengthwise, a braided, nonabsorbable, FiberWire-type (super suture), is optimal [19,23,29]. 

In pediatric patients, the average age of injury is in adolescence. Skiing, sport, and motor vehicle accidents are now increasing modes of injury, surpassing the traditional fall off of a bicycle. One possible explanation is that over the years, the amount of children involved in sports from early ages has significantly improved. In the USA, Martens (1998) estimated from a variety of sources that between the ages of 6 and 18 years old, 20/45 million youths (44%) were involved in non-school sports. More recently, Katzmarzyk et al., 2018 summarized the results of the United States Report Card on Physical Activity for Children and Youth: about 54% of high school students were involved in at least one sport, 56% of 6–12 years old children affirmed participating in team or individual sports, and approximately 77% of 6 to 17 years old live in a place surrounded by at least a park or a playground area [30,31]. For this reason, strategies in prevention of injury in sports is mandatory. General implementation of higher equipment technology, awareness, and a more staged learning curve is key [32,33].

If closed reduction is impossible in type II or III fractures, this may be due to entrapment of the anterior horn of the lateral or medial meniscus, or transverse ligament [12,34]. Secondary osteoarthritis that could lead to an overstress and to the rupture of the cartilage tissue is at low risk to develop after TSAF repair, and the knee functionality is re-established [35,36,37]. 

The use of osteoinductive agents in fracture surgery is still being studied and clinical indications are continuously evolving. Bone morphogenic proteins (BMPs) are potent osteoinductive agents and members of the transforming growth factor β family. In the treatment of tibial plateau fractures, the use of osteoinductive and osteoconductive agents may lead to better structural integrity and promote healing. However, Boraiah et al. found out that the incidence of heterotopic ossification was significantly higher in the BMP-positive patients than in the BMP-negative ones. Being given this, the use of BMP in periarticular fractures can lead to undesired reinterventions, so the caregivers should balance first the pearls and the pitfalls of these products before utilizing them [38,39].

There are data that confirm that residual laxity and intrinsic damage at the time of TSAF can lead to instability events and postponed ACL ruptures. Mitchell et al., through their cohort of 19 patients, stated that some of the children that suffered from TSAF will require later ACL reconstruction that is more significant when they fully develop the musculoskeletal integrity. A recent study by Murray et al. has shown that Bridge-Enhanced ACL repair (BEAR) in adolescents and young adults is not inferior to autograft anterior cruciate ligament reconstruction after a 2-year follow-up in terms of anteroposterior knee laxity and superior hamstring muscle strength. Further study should be performed to sustain these encouraging findings [40,41]. 

Arthroscopy is using only two portals and enables for full evaluation of the joint regarding the involvement of other structures and concomitant injuries. It also enables early mobilization, rapid rehabilitation, and fewer days of hospitalization. Arthroscopic treatment comprises fixation with either suture or cannulated screws and ACL reconstruction. As stated before, the limitations of this technique reside in the difficulty in inserting the suture wire, the low tension, the lack of proper reduction, and the fact that the tunnels that may pass through the fracture can affect the full reduction of the fragment. Simultaneous injuries that occur with TSAF are knee ligament lesions such as medial collateral ligament rupture, meniscus tears, bone bruise, tibial plateau fracture, or concomitant ACL tears. Ishibashi et al. described that bone bruises are the most encountered associated lesion. However, these findings are more frequent in the adult population, rather than in children, mainly due to the higher amount of mechanical force required to provoke tibial spine fractures [42]. 

The main limitation of the study resides in the sample size. Although there is a relatively low number of patients included, the TSAF are not commonly encountered, so the results are clinically significant.

## 5. Conclusions

Arthroscopic or open reduction and internal fixation for type II and III fractures is promoted in the current literature, despite a few case series of closed reduction of these fractures that demonstrated no difference in effect. With novel arthroscopic and fixation methods, the perks of surgery include restoring the ACL, salvaging interposed soft tissue, lowering the risk of malunion, and potentially reducing the time of immobilization to minimize stiffness. Patient selection is a major factor of success for this technique. Preoperative planning with computed tomography interpretation is essential. For patients with difficult proximal tibial fractures, the arthroscopic approach, used with experience, can decrease the trauma, provided that strong fixation consistent with early recuperation is done. The study provides encouraging results in terms of fracture healing, knee stability, and functional scores.

## Figures and Tables

**Table 1:** Pros and cons of arthroscopic wire suture technique.

Pros	Threats
Only two portals are used	The tunnels may pass through the place of fracture and this can alter complete reduction of the fragment
Arthroscopic use allows management of associated injuries	Difficulty in inserting the suture wire
No need for expensive implants	Low tension, not enough reduction

**Table 2:** Demographic data and patient characteristics.

Characteristic	
Gender	
Male, no. (%)	4 (33)
Female, no. (%)	8 (66)
Age, years, mean ± SD	14.3 ± 2.1
Professional athlete, yes, no. (%)	5 (42)
Time from the trauma, weeks, mean ± SD	2.1 ± 1

**Table 3:** Outcomes before and at each follow-up.

Variable	Preoperative	3 * mo. Follow-Up	6 mo. Follow-Up	** p-Value
IKDC, mean, ± SD	33.4 ± 23.3	61.1 ± 11.2	84.2 ± 14.3	<0.01
Tegner, mean, ± SD	3.8 ± 1.1	3.9 ± 0.9	6.7 ± 2.2	<0.02
Lysholm, mean, ± SD	53.7 ± 17.3	67.3 ± 9.4	87.7 ± 9.9	<0.01

* mo.—months, ** calculated using preoperatively and 6 months final follow-up outcomes.
